# Anomalous course and stenosing tenosynovitis of the extensor pollicis longus tendon at the radial styloid process: A case report

**DOI:** 10.1097/MD.0000000000012128

**Published:** 2018-09-14

**Authors:** Young-Keun Lee, Malrey Lee

**Affiliations:** a Department of Orthopedic Surgery, Research Institute of Clinical Medicine of Chonbuk National University—Biomedical Research Institute of Chonbuk National University Hospital; b The Research Center for Advanced Image and Information Technology, School of Electronics & Information Engineering, Chonbuk National University, Jeonju, Chonbuk, Republic of Korea.

**Keywords:** anomaly, de Quervain's disease, extensor pollicis longus, tenosynovitis

## Abstract

**Rationale:**

Anomalous course and tenosynovitis of extensor pollicis longus (EPL) tendon is a rare condition that presents clinical symptoms very similar to de Quervain's disease. Herein we report a case of anomalous course and tenosynovitis of the extensor pollicis longus (EPL) tendon associated with symptoms of de Quervain's disease.

**Patient concerns:**

A 44-year-old right-handed man visited the clinic because of radial pain associated with the left wrist, which was aggravated during the previous 10 days. The patient tested positive on the Finkelstein's test and displayed a limited range of motion of the left wrist. Motion of the thumb and wrist aggravated pain.

**Diagnoses:**

Magnetic resonance imaging (MRI) of the left wrist suggested mild tenosynovitis at the third extensor compartment and intersection syndrome. However, clinical symptoms failed to match the MRI findings.

**Interventions:**

A zig-zag skin incision on the radial styloid process was made. The operative findings revealed stenosing tenosynovitis with partial tearing. Retraction of the tendon extended the thumb interphalangeal joint, suggesting that the tendon was the EPL tendon rather than EPB tendon. After operation, we reviewed the MRI of the patient, which revealed that the oblique course of the EPL tendon originated from the ulnar side of the forearm to the radial styloid at the radial and proximal site of Lister's tubercle. No EPB tendon was present.

**Outcomes:**

At 12 months of follow-up, the patient's radial styloid process was completely asymptomatic and resumed full daily activities.

**Lessons:**

Anomalous course of the EPL tendon is rarely reported associated with similar symptoms of de Quervain's disease. However, the knowledge and understanding of this potential anomaly in the course of EPL tendon is very important for the treatment of de Quervain's disease to decrease patient dissatisfaction after surgery.

## 1 Introduction

De Quervain's disease is a stenosing tenosynovitis of the first dorsal extensor compartment of the wrist.^[1]^ This compartment is located over the styloid process of the radius, contains the abductor pollicis longus (APL) and the extensor pollicis brevis (EPB) tendons. The clinical presentation is fairly consistent and the diagnosis is made without difficulty following a complaint of several weeks’ or months’ history of pain localized along the radial side of the wrist, with exacerbation of symptoms upon movement of the thumb. The findings of local tenderness and swelling around the radial styloid and a positive Finkelstein's test are pathognomonic for de Quervain's disease.^[2]^ Several theories exist regarding the etiology of de Quervain's disease, including aberrant tendons and anatomic variations of the tendons and their sheaths.^[3,4,5]^ However, it is a rare condition that presents clinical symptoms very similar to de Quervain's disease due to the anomalous course and tenosynovitis of extensor pollicis longus (EPL) tendon despite few published reports.^[6,7,8]^

We report a case of an anomalous course and tenosynovitis of the EPL tendon, presenting with symptoms of de Quervain's disease.

## 2 Consent

The patient signed informed consent for the publication of this case report and any accompanying images. The ethics committee of Chonbuk National University Hospital waived ethical approval of this study because it was a case report comprising fewer than 3 patients.

## 3 Case report

A 44-year-old right-handed man visited the clinic because of radial-side pain associated with the left wrist, which was aggravated during the previous 10 days. Pain in the left wrist started 4 months ago, during exercise in a gym. He reported that he was self-employed and played golf and underwent weight training. With regard to the wrist pain, the patient was diagnosed with de Quervain's disease at another clinic, and was treated with physical therapy, nonsteroidal anti-inflammatory drugs and steroid injections twice, with temporary pain relief. The patient had no evidence of systemic diseases or trauma history.

**Figure 1:**
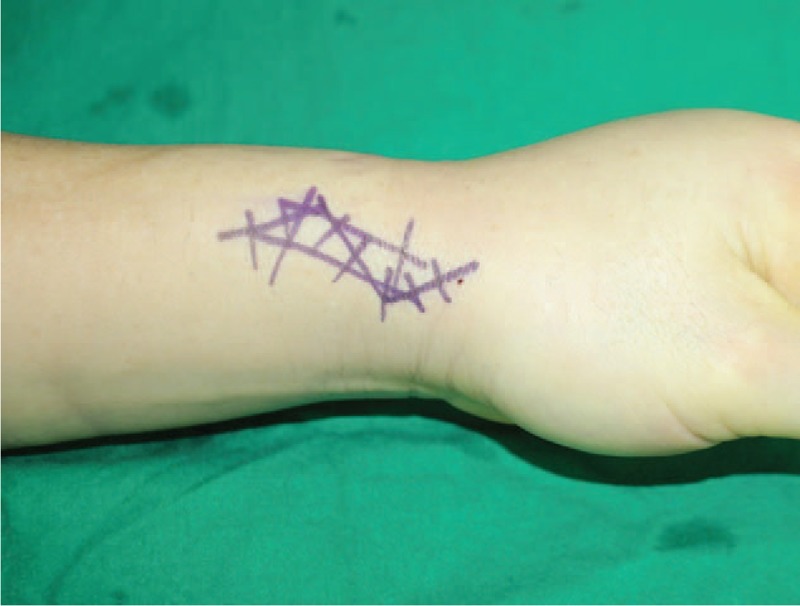
Initial photograph of the left wrist, with zig-zag incision lines over the radial styloid process.

**Figure 2:**
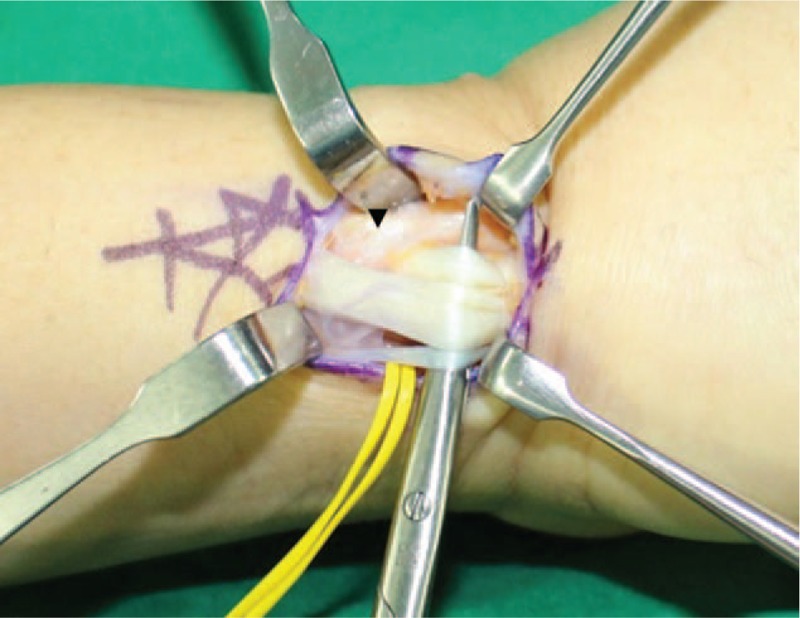
An intraoperative photograph of the left wrist after the division of retinaculum of the first extensor compartment showing 4 strands of extensor tendons and another extensor retinaculum over the ulnar most tendon (arrowhead).

**Figure 3:**
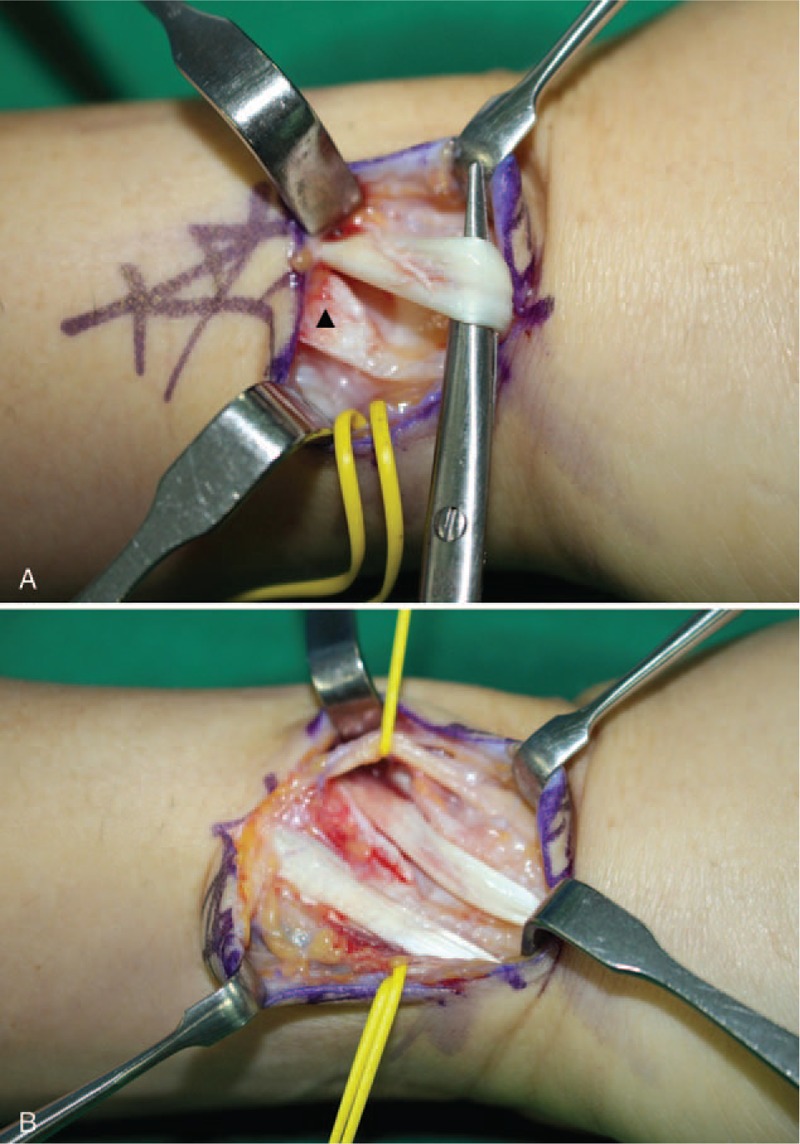
Intraoperative photograph of the left wrist after division of another retinaculum showing (A) stenosing tenosynovitis and partial tearing of the tendon. Hard bony protrusion (arrowhead) separated the course of tendon from previously exposed tendons. (B) View after removal of bony protrusion and debridement of tendon.

Upon examination, the patient's wrist showed mild swelling on the radial side, severe tenderness associated with the radial styloid process and moderate tenderness on the mid-dorsal aspect of the radio-carpal joint, which was occasionally swollen and inflamed. He yielded positive results in Finkelstein's test and displayed limited range of motion of the left wrist. Motion of the thumb and wrist aggravated the pain. The left-wrist grip strength was 18 kg (29 kg in the right) and thumb pinch strength was 5 kg (8 kg in the right). The visual analog scale (VAS) score for pain was 10 at rest. Initial plain left-wrist radiograph showed no abnormalities. Magnetic resonance imaging (MRI) of the left wrist at another clinic suggested mild tenosynovitis at the third extensor compartment and intersection syndrome. However, the clinical symptoms failed to match the MRI findings. Therefore, we clinically diagnosed him with de Quervain's disease and focal synovitis of radio-carpal joint. We decided to perform diagnostic wrist arthroscopy for the radio-carpal joint and retinacular release of the first extensor compartment for de Quervain's disease. Wrist arthroscopy using standard portals under general anesthesia yielded the following arthroscopic findings: dorsal synovitis, scapho-lunate and lunato-triquetral instability. Upon completion of the arthroscopic procedure, a zig-zag skin incision was made on the radial styloid process (Fig. 1). The superficial radial nerve was isolated followed by volar retraction and release of the retinaculum of the first extensor compartment. The APL tendon presented in 3 slips and the condition of APL tendon was not bad relatively, although we failed to find EPB tendon. Therefore, we incised the skin more distally, followed by location of another tendon ulnar to the APL tendon at a more distal site, separated by another retinaculum (Fig. 2), which was released. The operative findings revealed stenosing tenosynovitis with partial tearing (Fig. 3A). We initially considered the tendon to be the EPB tendon. However, the course of the tendon was unusual, warranting a further skin incision proximally, but not to the Lister's tubercle, to trace the course of the tendon. Hard bony protrusion instead of a septum separated the 2 tendons clearly (Fig. 3B). We partially removed the hard bony protrusion. Retraction of this tendon extended the thumb interphalangeal joint, suggesting that this tendon was the EPL tendon instead of EPB tendon.

Upon completion of the operation, we reviewed the MRI of the patient. It revealed that the oblique EPL tendon originated from the ulnar side of the forearm to the radial styloid at the radial and proximal sites of Lister's tubercle (Fig. 4 A and B). No EPB tendon was found. During the follow-up, we could not find the anatomical snuff box in his left wrist. However, in his healthy right wrist, the EPL tendon ran ulnar to Lister's tubercle in a normal course and the anatomical snuff box was recognized on physical examination (Fig. 5).

**Figure 4:**
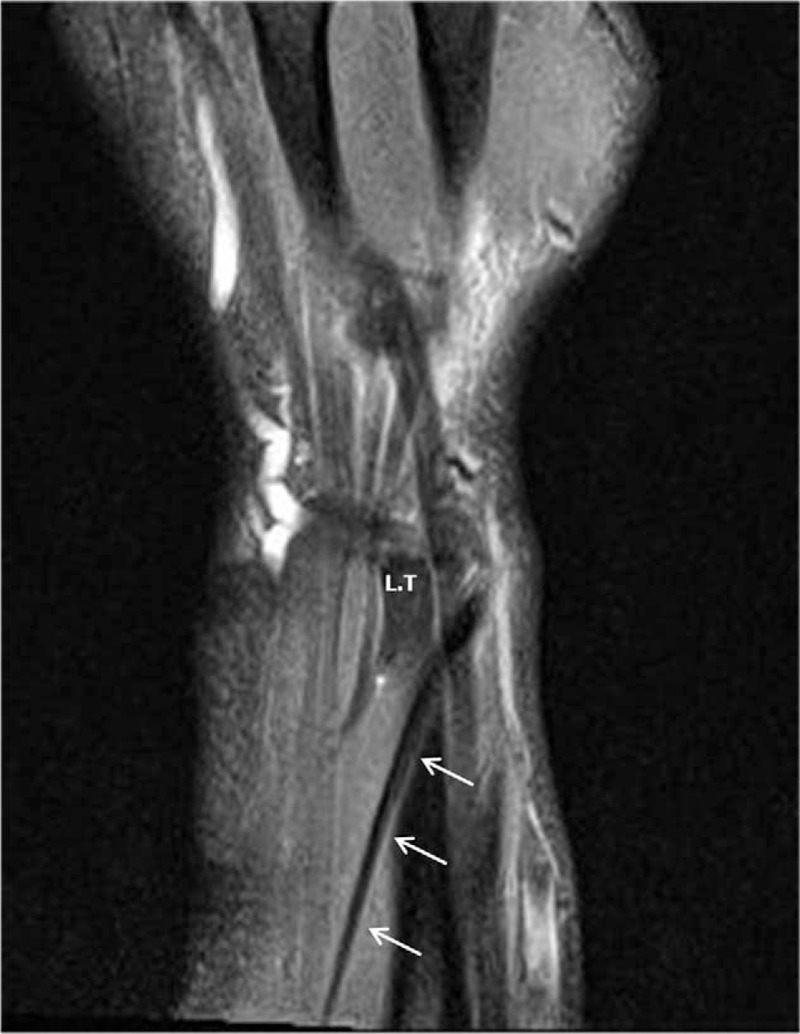
Coronal (A) and axial (B) fat-suppressed T2-weighted images at the left wrist level showing the oblique course of EPL tendon, which originated in the ulnar side of the forearm and extended to radial styloid at the radial and proximal sites of Lister's tubercle. (Arrows indicate the EPL tendon, LT: Lister's tubercle). EPL = extensor pollicis longus.

**Figure 4 (Continued):**
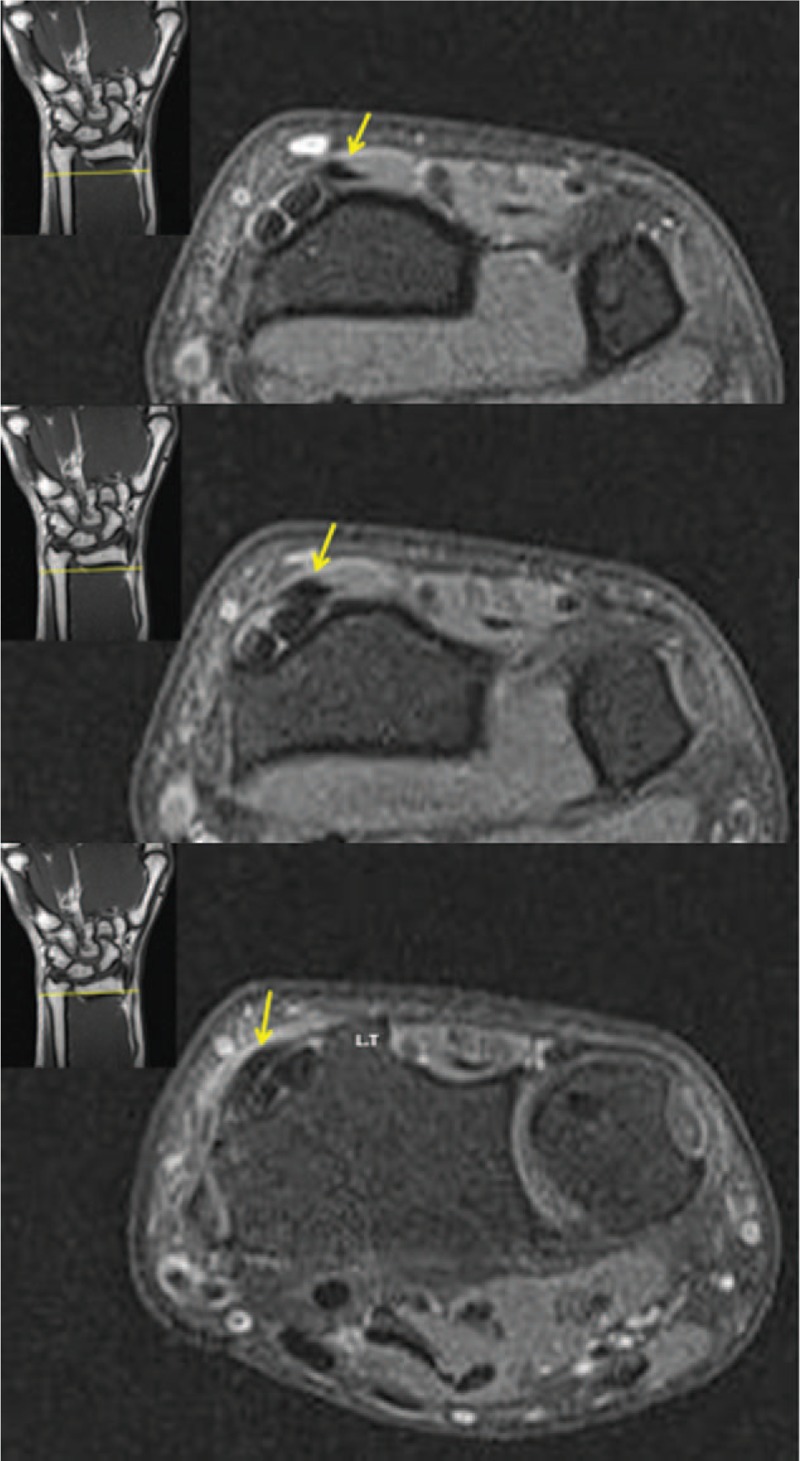
Coronal (A) and axial (B) fat-suppressed T2-weighted images at the left wrist level showing the oblique course of EPL tendon, which originated in the ulnar side of the forearm and extended to radial styloid at the radial and proximal sites of Lister's tubercle. (Arrows indicate the EPL tendon, LT: Lister's tubercle). EPL = extensor pollicis longus.

**Figure 5:**
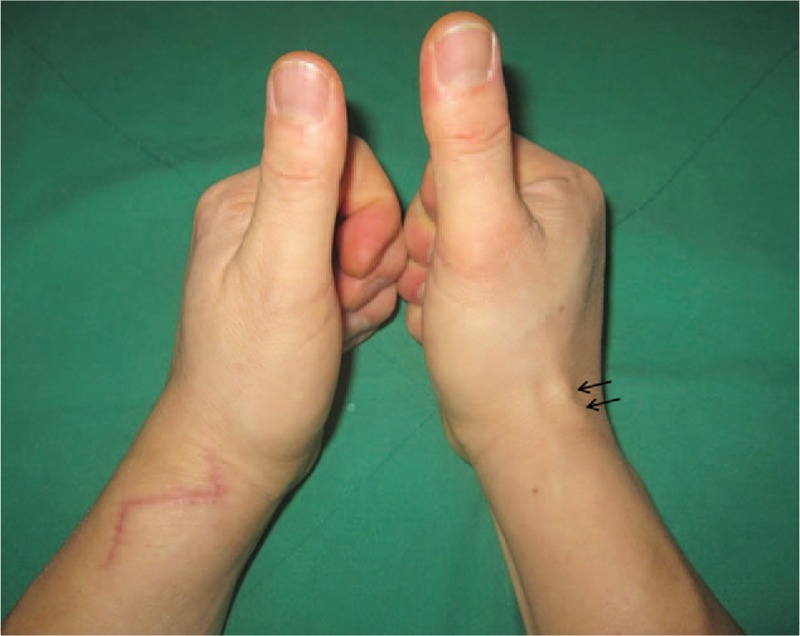
Follow-up photograph showing the normal course of EPL tendon (arrows) and anatomical snuff box in the right wrist. EPL = extensor pollicis longus.

At 12 months follow-up, the patient was completely asymptomatic at the radial styloid process and able to resume full daily activities.

## 4 Discussion

The anatomic variations of the first dorsal extensor compartment may have an effect on the underlying pathophysiology of de Quervain's tenosynovitis and be associated with a relatively poorer response to nonsurgical treatment.^[1,4]^ Bahm et al^[4]^ reported a division of the first dorsal compartment by an additional septum in 60% of patients with symptomatic de Quervain's disease. The APL tendon consisted of multiple tendons in 76% of the patients. The EPB tendon included a single tendon in 96% of the patients. Gao et al^[9]^ reported an incidence of 67.5% for general septum in 40 cadavers, and the mean length of the septum was 5 mm. Therefore, during surgery, the sheath covering the first dorsal compartment as well as a separate subcompartment for the 2 tendons should be released. It was reported that exploring 5 mm proximal from the radial styloid process was especially useful for identifying the septum and the subcompartment.^[9]^ Missing this step is the most common cause of dissatisfaction after the release of first dorsal compartment for de Quervain's tenosynovitis.^[10]^ We also evaluated other subcompartments after releasing the retinaculum of the first dorsal compartment. However, initially due to detection challenges, we dissected more distally to the radiocarpal joint. We found another slip of tendon in the ulnar side of APL tendon. Surprisingly, it was EPL tendon. Although some studies have reported to have found an accessory EPL tendon during anatomical dissection, the EPL tendon is known to be a consistent structure, unlike the anomalies and variations of other extensor tendons of the hand.^[6,11,12]^

Anomalous course of EPL tendon with similar symptoms of de Quervain's disease is extremely rare. To date only 5 cases of anomalous course of EPL tendon in symptomatic patients have been reported in the English literature.^[6–8,12]^ Nishijo et al^[6]^ described duplicated EPL passing radial to Lister's tubercle into the first dorsal compartment and in her left hand, ulnar to Lister's tubercle in a normal course. Sawaizumi et al^[12]^ also described the anomalous course of duplicated EPL tendon, one of them in a new compartment. Abe et al^[7]^ described 2 cases with a single EPL tendon. They also described the failure to detect the anatomical snuff box in the healthy wrist in the 2 cases. Rubin et al^[8]^ described single EPL tendon running radial to Lister's tubercle into the first dorsal compartment with symptoms resembling intersection syndrome initially and de Quervain's disease 9 months later. In our case, a single EPL tendon extended radial to Lister's tubercle into a new compartment between the first and the second compartments with symptoms of de Quervain's disease. But we could find the anatomical snuff box and the normal course of EPL tendon in the patient's healthy right wrist, unlike the results of Abe et al.^[7]^

Stenosing tenosynovitis of EPL tendon is very rare in the nonrheumatoid patient although it was more common in drummers or following trauma to the wrist.^[13]^ Presenting symptoms include pain, swelling and crepitation in Lister's tubercle; pain is aggravated by active or passive thumb motion. Surgical treatment is indicated for patients with tenosynovitis of the EPL tendon if conservative treatments are unsuccessful. A study by Kardashian et al^[14]^ recommended that the surgeon should be prepared to perform either free tendon grafting or an extensor indicis proprius to EPL transfer, as the EPL tendon might be rupture or markedly attenuated by the time of surgery. In our case, however, the symptoms were predominantly in the radial styloid process, eluding the diagnosis of tenosynovitis of the EPL tendon at the first examination.

The limitation of this study is that the final follow-up of patient was conducted as a phone interview, which prevented examination of their clinical photos and plain radiographs. It is expected that the surgeons will take into consideration the possibility of an anomalous course of the EPL tendon in patients with suspected de Quervain's disease, and will examine the anatomical snuff box of the patient to confirm the presence of the EPL tendon.

## 5 Conclusion

Anomalous course of the EPL tendon is rarely associated with similar symptoms of de Quervain's disease. However, we considered that the knowledge and understanding of this potential anomaly in the course of EPL tendon is critical to the treatment of de Quervain's disease and decrease patient dissatisfaction after surgery.

## Author contributions

**Conceptualization:** Malrey Lee.

**Writing – original draft:** Young Keun Lee, Malrey Lee.

**Writing – review & editing:** Young Keun Lee, Malrey Lee.
